# Ceramides As Potential New Predictors of the Severity of Acute Coronary Syndrome in Conjunction with SARS-CoV-2 Infection

**DOI:** 10.32607/actanaturae.27400

**Published:** 2024

**Authors:** N. G. Lozhkina, O. I. Gushchina, N. V. Basov, E. V. Gaisler, A. D. Rogachev, Yu. S. Sotnikova, Yu. V. Patrushev, A. G. Pokrovsky

**Affiliations:** Novosibirsk State University, Novosibirsk, 630090 Russian Federation; Federal Research Center for Fundamental and Translational Medicine, Novosibirsk, 630117 Russian Federation; City Clinical Hospital No. 1, Novosibirsk 630047 Russian Federation; Vorozhtsov Novosibirsk Institute of Organic Chemistry, Novosibirsk, 630090 Russian Federation; Boreskov Institute of Catalysis, Novosibirsk, 630090 Russian Federation

**Keywords:** acute coronary syndrome, myocardial infarction, SARS-CoV-2 infection, metabolomics, ceramides

## Abstract

Acute coronary events (ACEs) associated with a SARS-CoV-2 infection can significantly differ from classic ACEs. New biomarkers, such as ceramides, may help in the diagnosis and treatment of this disease. This study included 73 ACE patients for whom the SARS-CoV-2 infection was verified. Two subgroups were formed: the favorable outcome subgroup and the fatal outcome subgroup. Plasma samples were collected from all patients at the time of admission for a metabolomic analysis. The analysis of metabolites revealed that the ceramide levels were significantly lower in the fatal outcome subgroup than in the survivor subgroup. Therefore, determining ceramide levels in patients with ACEs in conjunction with COVID-19 may help assess the prognosis of these patients and manage their risks.

## INTRODUCTION

Acute coronary events (ACEs) in conjunction with the SARS-CoV-2 infection can significantly differ from the classic manifestations of this disease. Many symptoms characteristic of a severe viral infection mask the manifestations of acute coronary syndrome. In turn, ACEs can also hide the signs of infection. Respiratory distress, high activity of inflammatory markers, chest pain, and, in severe clinical cases, shock and hypotension are difficult to differentiate at the starting point of their development. One of the major problems in making a clinical diagnosis is the late symptoms of the disease, including the delayed conversion of myocardial necrosis markers. For example, the titers of high-sensitivity troponin in myocardial infarction attain diagnostic significance 4 h after the onset of symptoms [1]. New biomarker sets may be useful in early diagnosis and in choosing the treatment modality. Recently, metabolomics-based strategies have been used to identify the molecular mechanisms involved in cardiovascular diseases.

Metabolomics technologies enable the identification, quantification, and characterization of low molecular weight metabolites weighing less than 1,500 Da [2]. Determination of the metabolomic profile of patients and identification of potential biomarkers may be helpful in early diagnosis of diseases and applications of personalized therapy.

Ceramides are a promising class of signaling molecules. This subclass of lipid molecules constitutes the hydrophobic backbone of all complex sphingolipids (e.g., sphingomyelin (SM), cerebrosides, gangliosides) and structurally consists of an acyl substituent of variable carbon chain length linked to the amino group of a sphingoid base, typically sphingosine. Ceramides are important components of all cell membranes. The fatty acyl chains, usually saturated or monounsaturated, may contain an OH grouplinked to C2 or to the terminal carbon atom (α- and ω-hydroxy fatty acids, respectively) [3]. The value of ceramides as diagnostic markers is associated with their high stability at various temperatures (which is reflected in the ease of sampling, storage, and transportation of biological material). The results of studies of the relationship between ceramides and a cardiovascular pathology are contradictory. During the COVID-19 pandemic, these biomarkers were actively studied in patients with different types of infections, but their role in the pathogenesis, course, and prognosis of the acute coronary syndrome associated with the SARS-CoV-2 infection remains unclear. In this regard, assessing the role of a number of key metabolites, in particular ceramides, as potential new predictors of the severity of ACEs in conjunction with SARS-CoV-2 infection seems topical.

## EXPERIMENTAL

**Research methods and characterization of patients**

The study included 73 patients who were consecutively admitted to the regional vascular center No. 1 of the Novosibirsk City Clinical Hospital No. 1 with a diagnosis of acute coronary syndrome (confirmed according to Russian and European clinical guidelines) in whom the SARS-CoV-2 infection was verified (no more than 28 days before or within 14 days after the onset of ACEs). All patients underwent a full range of examinations in accordance with current clinical guidelines for both pathologies: a complete blood count and biochemistry panel, a coagulogram with D-dimer levels, a PCR test for COVID-19, electrocardiography (ECG), echocardiography (EchoCG) at admission, computed tomography of the chest (chest CT), and coronary angiography (CAG) with percutaneous transluminal balloon angioplasty (PTCA) and stenting of the infarct-related artery using modern certified medical equipment [4, 5]. In addition to the standard examination, plasma samples were collected from all patients at the time of admission and frozen at –70°C for a metabolomic analysis that was performed at the Novosibirsk State University. The study protocol was approved at a meeting of the local ethics committee.

**Inclusion criteria**

Males and females aged 18 to 90 years admitted to the clinic with a diagnosis of acute coronary syndrome (with and without ST segment elevation) confirmed by a typical clinical picture, ECG, selective coronary angiography, and quantitative troponin I determination; a verified diagnosis of the SARS-CoV-2 infection (no more than 28 days before or within 14 days after the onset of an acute coronary event); signed voluntary informed consent.

**Exclusion criteria**

Lack of signed voluntary informed consent. The study did not include patients with malignant neoplasms, severe autoimmune diseases, terminal somatic pathology (liver cirrhosis of any severity, chronic kidney disease ≥ S4, patients on long-term hemodialysis), and pre-existing mental disorders at baseline.

**Study design**

This was an open, continuous, prospective, non-randomized, and parallel group study that included patients with acute coronary syndrome and a verified new coronavirus infection who were consecutively admitted to the emergency cardiology department of City Clinical Hospital No. 1 in 2021–2023. The diagnosis of ACE was established using a set of criteria developed by the European and Russian Societies of Cardiology (2020), which were as follows: a) clinical signs or symptoms of myocardial ischemia; b) ECG changes in two or more consecutive leads for acute ST-elevation myocardial infarction (STEMI) (high-amplitude T wave, negative T wave, ST segment elevation, pathological Q wave, ST segment depression, presence of QR). The diagnosis of a novel coronavirus infection was made according to temporary guidelines for the prevention, diagnosis, and treatment of a novel coronavirus infection (version 13 of October 14, 2021), which included a) a positive result of a SARS-CoV-2 RNA test using a nucleic acid amplification technique (NAAT) or immunochromatographic SARS-CoV-2 antigen testing; b) a high clinical probability (pulmonary CT, clinical picture, and relevant epidemiological history data) [4, 5].

**Sample collection, preparation, and analysis**

Blood samples were collected from patients on the day of hospital admission. Venous blood was sampled into vacuum tubes containing the K-EDTA anticoagulant. Plasma was obtained by centrifugation, transferred to a clean tube, and frozen at –80°C until sample preparation. Sample preparation was performed according to [6]. Blood plasma (100 μL) was added with 400 μL of a cooled methanol and acetonitrile mixture (1 : 1). Samples were shaken on a shaker and then centrifuged at 16 000 rpm and +4°C for 15 min. The supernatant was transferred into a glass vial insert and analyzed. Samples prepared by mixing equal volumes of patient plasma were used for quality control.

The metabolomic analysis was performed according to [7]. The HPLC-MS/MS analysis was performed on a Shimadzu LC-20AD Prominence chromatograph equipped with a gradient pump, a SIL-20AC autosampler (Shimadzu, Japan) thermostated at +10°C, and a CTO-10A Svp column thermostat at a temperature of +35°C. Chromatographic separation was performed on a monolithic column with a 1-vinyl-1,2,4-triazolebased sorbent, which was prepared according to the method in [8]. We used an aqueous (NH4)2CO3 solution (20 mM) containing 5 vol.% acetonitrile and adjusted to a pH of 9.8 with a 25% ammonia solution as mobile phase A; mobile phase B was pure acetonitrile. The reversed phase chromatography gradient was as follows: 0 min, 0% B; 1 min, 0% B; 6 min, 98% B; 16 min, 98% B, after which the column was equilibrated for 3 min. The hydrophilic chromatography (HILIC) gradient was as follows: 0 min, 98% B; 2 min, 98% B; 6 min, 0% B; 10 min, 0% B, after which the column was equilibrated for 4 min. The flow rate was 300 µL/min, and the sample volume was 2 µL.

Metabolites were detected on an API 6500 QTRAP mass spectrometer (AB SCIEX, USA) equipped with an electrospray ionization source operating in positive and negative ionization modes. Metabolites were detected in the multiple reaction monitoring (MRM) mode. The main mass spectrometric parameters were as follows: the ion spray (IS) voltage was 5,500 V for positive and –4,500 V for negative ionization; drying gas temperature was 475°C; collision cell gas (CAD) was “high”; gas 1, gas 2, and curtain gas pressures were 33, 33, and 30 psi (227.5, 227.5, and 206.8 kPa, respectively); the declustering potential (DP) was ±91 V; the entrance potential (EP) was ±10 V; and the collision cell exit potential (CXP) was ±9 V. Device control and data collection were performed using the Analyst 1.6.3 software (AB SCIEX, USA). The chromatograms were processed using the MultiQuant 2.1 software (AB SCIEX, USA).

The samples were divided into two groups: the favorable outcome (recovery) group and the in-hospital fatal outcome group. Samples from these groups were subjected to a metabolomic analysis, and key metabolites were identified. The difference between the two subgroups of “fatal” and “survived” patients were assessed using the Mann–hitney test. The critical value for subgroup dimension was MW_crit_ = 32.

## RESULTS

The selected groups differed significantly in age: the mean age in the first group was 63.6 ± 9.6 years and 73 ± 8.2 years in the second (unfavorable outcome group) (*p *= 0.003). Group 1 included 37 males and 24 females, and group 2 consisted of 5 males and 6 females. All patients who died had ACS with ST elevation; in the favorable outcome group, ST elevation was diagnosed in 56 patients, and ACS without ST elevation was diagnosed in 5 patients.

The severity of the SARS-CoV-2 infection was as follows: in the favorable outcome group, the infection course was mild and asymptomatic, moderate, or severe in 22, 26, and 12 patients, respectively; in the fatal outcome group, the SARS-CoV-2 infection course was asymptomatic, mild, moderate, or extremely severe in 0, 1, 1, and 9 patients, respectively.

Analysis of the clinical and laboratory parameters revealed significant differences between the study subgroups of patients: any form of atrial fibrillation was more common in the fatal outcome group than in the survivor group (*p* < 0.5); the serum iron level was lower in the unfavorable outcome group (*p* < 0.001), and albumin was significantly lower in the unfavorable outcome group than in the survivor group (*p* < 0.001). On the contrary, the D-dimer level was higher in group 2 (*p * < 0.0001). The mean C-reactive protein concentration on admission was significantly lower in group 1 than in group 2 (*p *= 0.0243). Indicators of myocardial contractility of both the left and right ventricles were significantly worse in the fatal outcome group (*p * < 0.0001). No significant differences were found in the degree of coronary artery lesion and lipid panel parameters. Thus, our clinical, laboratory, and instrumental data are consistent with the data of other researchers [9, 10].

**Table 1:** The values of the key metabolites in the study groups

Metabolite	MW*	Multiplicity lethal/non-lethal
Ceramide (d18:1/22:0)	4	0.503
Ceramide (d18:1/24:0)	9	0.531
Ceramide (d18:1/24:0 OH)	11	0.579
Ceramide (d18:1/22:2 OH)	12	0.564
Ceramide (d18:1/23:0) or ceramide (d18:1/22:1 OH)	12	0.529
Ceramide (d18:1/25:0)	13	0.524
Glycosphingolipid (18:1/22:0)	13	0.486
Glycosphingolipid (18:1/24:1)	15	0.356
Ceramide (d18:1/20:1 OH)	18	0.621
Ceramide (d18:1/22:0 OH)	18	0.685
Ceramide (d18:1/24:1)	19	0.450
Ceramide (d18:1/20:0)	21	0.658
Ceramide (d18:1/18:0)	23	0.695
Ceramide (d18:1/26:1)	23	0.702
Sphingomyelin (d18:1/22:0 OH)	23	0.663
Ceramide (d18:1/16:0 OH)	24	0.712
Ceramide (d18:1/26:2)	24	0.653
Ceramide (d18:1/16:1 OH)	25	0.731
Ceramide (d18:1/24:2 OH)	27	0.641
Sphingomyelin (d18:1/22:2)	27	0.683
3-Phosphoglyceric acid	28	0.456
Ceramide (d18:1/18:0 OH)	28	0.680
Sphingomyelin (d18:1/24:0)	28	0.569
Ceramide (d18:1/18:1 OH)	29	0.719
Ceramide (d18:1/18:1)	29	0.738
Corticosterone	29	0.594
Glycosphingolipid (18:1/20:0)	29	0.648
Sphingomyelin (d18:1/16:2 OH)	29	0.750
Sphingomyelin (d18:1/18:2 OH)	30	0.686
Plasmalogen (p18:0/22:6)	31	0.655
5-Hydroxyindoleacetic acid	32	1.788
Glycosphingolipid (18:1/16:0)	32	0.561

^*MW^ – Mann–Whitney U-statistic value.

At the next stage, key metabolites in the blood plasma of patients were identified. Comparison of the mean indicator values revealed “saturation” of the isolated metabolites with a group of ceramides (19 compounds, *Table 1*), as well as five metabolites from the sphingomyelin (SM) class and four metabolites from the glycosylceramide (GC) class.

**Fig. 1:**
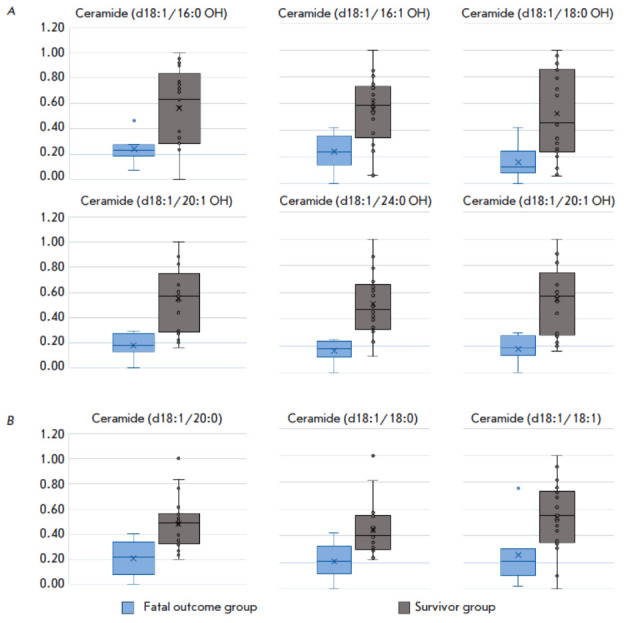
Plasma ceramide levels in the study groups. (*A*) Hydroxylated ceramides; (*B*) Non-hydroxylated ceramides

Comparative analysis of the levels of the identified metabolites in the samples showed that the plasma levels of all metabolites in fatal outcome patients were noticeably lower than those in survived patients. The only exception was 5-hydroxyindoleacetic acid, whose level increased more than 1.5-fold. *Figure* shows the normalized peak areas of several ceramides in the two groups.

## DISCUSSION

Ceramides are involved in various cellular processes, including pathological ones. Their levels in resting cells are extremely low, but they can significantly increase under cellular stress or in response to various stimuli (cytokines, apoptosis receptor ligands, antitumor drugs). Furthermore, accumulating evidence indicates that the structural features of different ceramide species may underlie their specificity for certain cellular processes [11]. However, the molecular mechanisms that underlie this specificity and the mode of ceramide action on cells remain to be studied in detail. This mechanism is thought to be associated with changes in the biophysical properties of membranes which occur during ceramide formation. These changes are partially associated with the unique molecular structure of ceramides, in particular their very small functional groups, hydrophobicity, and high melting point, which reduces their miscibility with other membrane lipids. Several studies have reported increased membrane permeability associated with ceramide formation under the action of bacterial sphingomyelin phosphodiesterase (SMase) or addition of ceramides to preformed membranes. It is suggested that the formation of ceramides on the cell membrane may lead to changes in lipid–lipid, lipidprotein, and protein–protein interactions, which may significantly affect protein activity and thereby signaling processes [12].

There are studies on the role of ceramides in the development and progression of cardiovascular pathology. The development of atherosclerotic plaques is known to be a complex process, mainly associated with inflammation, which begins with endothelial damage and is accompanied by local invasion of immune cells, lipid accumulation, and vascular wall remodeling. Induction of cellular apoptosis was initially thought to be the main cause of ceramide-related cell damage [13]. But later, this suggestion was called into question because cellular ceramide levels were found to increase only at later apoptosis stages [14, 15]. In macrophages, endothelial cells, hepatocytes, and tumor cell lines, such as the MCF7 breast cancer cell line, ceramides have been shown to mediate the cellular effects of the tumor necrosis factor-α (TNF-α) receptor [16]. High ceramide levels were also found to be associated with myocardial cell death in a mouse myocardial infarction model. In addition, ceramides can cause vascular dysfunction via deactivation of endothelial NO synthase [17].

A reduction in ceramide levels in cells and tissues by inhibition of the enzymes involved in ceramide formation prevents the development of atherosclerosis in animal models [18]. In vascular tissues, ceramides are produced in response to hyperglycemia and TNF-α signaling and are involved in NO signaling and inflammation. Elevated ceramide levels in human blood are associated with cardiovascular events. In addition, cardiovascular risk factors, such as obesity and diabetes mellitus, are associated with ceramide accumulation [19].

One of the first studies linking blood ceramide levels and cardiovascular disease progression was conducted by Meikle et al. [20]. Since then, there have been observational studies clearly demonstrating the relationship between certain ceramide subtypes and an increased risk of cardiovascular events. The most comprehensive studies were conducted by the Hilvo Laaksonen groups [21, 22]. These studies were used for developing two different risk scores that demonstrate that, in particular, C16:0, C18:0, and C24:1 ceramides may be markers of high risk of cardiovascular events, which are independent of the other cardiovascular risk factors identified in patients with coronary heart diseases [21, 22]. It should be noted that a relationship between elevated C18:0 ceramide levels and major cardiovascular events was also present in patients without known coronary artery diseases and appeared independent of other cardiovascular risk factors.

Many studies have demonstrated the importance of ceramides in the inflammatory response. For example, ceramides are involved in pro-inflammatory signal transmission in endothelial cells [23]. In the cardiovascular system, inflammatory processes are activated by various stimuli; e.g., pathogen- or damageassociated molecular patterns [24]. Although the exact mechanisms underlying this phenomenon are not fully understood, several studies have demonstrated a correlation between ceramides and activation of inflammatory diseases. This was first reported by Koka et al., who used pharmacological inhibition of acid sphingomyelinase (ASM) by amitriptyline, as well as RNA interference (RNAi), to study endothelial cells of ASM−/− mice to show that ASM mediates the inflammatory response involving the inflammasome NLR family pyrin domain-containing protein 3 (NLRP3) [25]. These results were confirmed *in vivo *in ASM−/− mice and were also replicated in a study using RNAi against ASM in endothelial cells [26]. The role of ceramides in NLRP3 activation in macrophages is less clear. Camell et al. [27] did not find that the *de novo* synthesis pathway, which involves serine palmitoyltransferase, participates in the activation of inflammation. However, other pathways of ceramide production were not analyzed. Scheiblich et al. showed that SPT activation or external application of non-physiological C2 ceramide leads to NLRP3 activation and interleukin-1β (IL-1β) release in microglial cells [28]. Administration of ceramide C2 led to the activation of inflammation in bone marrow-derived macrophages [29]. It has been suggested that ceramides and inflammation activation are related [30, 31]. In particular, ceramides produced in reaction to ASM appear to be important for inflammatory signaling [32]. Finally, it remains unclear whether ceramides are directly activated during inflammatory processes or if activation is mediated by pathogen- or damage-associated molecular patterns. Evidence of direct activation of inflammation by ceramides is currently lacking.

**Ceramides and SARS-CoV-2**

Research has demonstrated that ceramide levels can be both elevated and decreased in SARS-CoV-2. Elevated ceramide levels may be associated with the activation of apoptosis, which leads to cell death and probably promotes inflammatory processes typical of severe forms of COVID-19. On the other hand, a decrease in ceramide levels may be associated with a depletion of their precursors or disruption of their synthesis by the virus [33, 34]. Although the mechanism of binding of the SARS-CoV-2 virus to its receptor [35, 36], angiotensin-converting enzyme 2 (ACE2), and TMPRSS2 protease, which activates viral polymerase, is well understood, changes in the cell membrane during infection are a complex and multifactorial process. Virus processing in the host cell is accompanied by significant changes in the membrane lipid composition, in particular changes in the levels of ceramides and other sphingolipids. These changes can be caused not only by apoptosis, but also by the virus that is able to alter membrane composition to optimize its replication, affecting the levels of ceramides and other lipids; disruption of the normal lipid metabolism in the cell, which can lead to changes in the levels of ceramides and other lipids. These pathological processes are involved in microvascular damage in SARS-CoV-2 and are, therefore, associated with cardiovascular complications in SARS-CoV-2 patients.

## CONCLUSIONS

The present study examined a unique disease phenotype – a conjunction of acute coronary syndrome with SARS-CoV-2. Comparison with ACEs without SARS-CoV-2 revealed increased ceramide levels in the group of ACEs without SARS-CoV-2, which may indicate that they play a role in the pathogenesis of this disease combination. In addition, there was a paradoxical response of the body’s metabolic system to an acute coronary event in conjunction with COVID-19: ceramide levels were significantly lower in the fatal outcome subgroup than in the survivor subgroup. The low ceramide levels in fatal outcome patients may be explained by a depletion of the precursors of these metabolites in the terminal condition, which may be due to the influence of the non-structural SARS-CoV-2 proteins that activate the metabolic pathways involved in apoptosis and inflammation. Also, active production of viral particles may lead to cellular exhaustion and destruction of the cell membrane, which may explain the unusually high levels of cell membrane components in the plasma of SARS-CoV-2 patients. But this phenomenon requires further study.

Therefore, this pilot study has showed that metabolomic profiling with a focus on ceramide levels may help assess the risk of a fatal outcome in patients with acute coronary syndrome in conjunction with the SARS-CoV-2 infection. Our findings need confirmation in other patient populations.
